# Analysis of Orthopaedic Surgery Residency Match Results: 2016 to 2022

**DOI:** 10.7759/cureus.75203

**Published:** 2024-12-06

**Authors:** Olivia B de Araujo, Daniel J O'Connell, Austin J Allen, Stuart L Mitchell

**Affiliations:** 1 Orthopaedic Surgery, Jackson Memorial Hospital, Miller School of Medicine, University of Miami, Miami, USA; 2 Orthopaedic Surgery, Geisinger South Wilkes-Barre, Wilkes-Barre, USA; 3 Neurological Surgery, Atrium Health Carolinas Medical Center, Charlotte, USA; 4 Orthopaedic Surgery, University of North Carolina at Chapel Hill School of Medicine, Chapel Hill, USA

**Keywords:** match, nrmp, orthopaedic residency match, orthopaedics, orthopaedic surgery

## Abstract

*Background*: Objective data examining match trends and applicant characteristics are in high demand given the stakes involved with the orthopaedic surgery match. Our study sought to analyse the trends among matched orthopaedic surgery applicants to clarify how medical students can best prepare for and assess their chances of a successful orthopaedic residency match.

*Methods*: We used the publicly available National Residency Matching Program (NRMP) *Interactive Charting Outcomes in the Match *tool to gather characteristics for matched and unmatched orthopaedic surgery applicants from 2016 to 2022 and compare the probability of matching for all applicants versus each subgroup of reported applicant characteristics using risk difference (RD) and two-tailed p-values.

*Results*: Step 1 score >250 (p <0.001), being a US MD applicant (p <0.001), ranking ≥11 contiguous programs (p<0.001), Step 2 score >250 (p <0.001), five to 10 research experiences (p <0.001), five to 15 publications (p <0.002), five to 25 volunteer experiences (p<0.03), and Alpha Omega Alpha (AOA) Honor Medical Society membership (p<0.001) were associated with increased chance of matching while having a non-PhD graduate degree decreased probability of matching (RD -8.3%, p<0.001) and a PhD degree had no associated change in match probability.

*Conclusions*: These results show that applicants who possess higher Step 1 and 2 scores, are AOA members, US MD applicants, and have a rank ≥11 in orthopaedic programs are more likely to successfully match. A continuing significant increase in the probability of matching was noted until more than 25 programs were ranked.

## Introduction

The orthopaedic surgery residency match has continued to increase in competitiveness, with an especially difficult application cycle in 2022, resulting in a 62.3% match rate - a 8.6% decrease from 2021 [1]. Despite a relatively stable match rate over the past decade, mean United States Medical Licensing Examination (USMLE) scores and the number of research experiences have continued to increase, demonstrating an increasing level of academic accomplishment among successful orthopaedic surgery applicants [2]. Since the size of the applicant pool has only increased by a factor of 1.2 from 1984 to 2020, while the average number of programs each applicant applies to nearly doubled between 2001 and 2018 [3], some studies have sought to explain increased competition as a misconception due to the increased number of applications submitted per candidate and subsequent importance programs placed on test score thresholds to filter through this increasing number of applications [4]. Other studies on both the orthopaedic match and that of other competitive subspecialties found applicant characteristics that consistently correlate with successful matches, such as honors grades in clinical clerkships, high test scores, Alpha Omega Alpha (AOA) Honor Medical Society status, and an increasing number of contiguous ranks [5-7].

The Association of American Medical Colleges (AAMC) publishes free, publicly accessible National Resident Matching Program (NRMP) data each year, which can be explored using the online Interactive Charting Outcomes in the Match (ICTO) [8] tool to compare the matched and unmatched applicant characteristics. Despite the availability of NRMP data [9,10], few publications have analyzed these data in recent years, particularly since osteopathic and allopathic residencies were combined and interviews were made virtual [6,11,12]. Furthermore, competitive surgical subspecialty applicants are increasingly dissuaded from “interview hoarding”, that is, accepting as many interviews as are offered and subsequently ranking as many programs as possible to increase chances of matching [13,14]. Though accepting as many interviews as possible may maximize an individual’s chance of a successful match, it may impair the match process overall.

This project builds on previous studies by providing statistical analysis of the orthopaedic surgery match from 2016 to 2022 to rank the importance of various applicant characteristics and illustrate trends in orthopaedic surgery match results. In clarifying which factors contribute to a successful match, this study enables medical students to make more informed decisions when considering how to invest their time when preparing to apply for orthopaedic residency.

This article was previously presented as a meeting abstract at the 2023 Eastern Orthopaedic Association Annual Meeting (October 2023), Medical Student Orthopaedic Society 2023 Symposium (April 2023), and the Orthopaedic Research Society Annual Meeting (February 2023).

## Materials and methods

We used the NRMP ICTO [8] tool to obtain data on the characteristics of matched and unmatched orthopaedic surgery applicants from biennial reports from 2016 to 2022 [15-18]. Data from the ICTO tool or extrapolated from published graphs were collected, stored, and analyzed using Excel version 16.68 (Microsoft, Redmond, WA). Reported data was stratified by applicant type (US senior medical students versus international medical graduates [IMGs]), medical school attended (DO versus MD), United States Medical Licensing Examination (USMLE) Step 1 and Step 2 scores, contiguous ranks, specialties ranked, research experiences, publications, work and volunteer experiences, having obtained a PhD or other non-doctoral graduate degree, and Alpha Omega Alpha Honor Society (AOA) membership status.

Contiguous ranks, defined as the number of programs ranked within one specialty by an applicant, are often used as a proxy for interviews accepted while specialties ranked represented the number of different specialties a given applicant applied into. Risk difference (RD) with 95% confidence intervals was tabulated to compare the match rate for each characteristic subgroup with the overall average. Paired t-tests were used to detect significant differences between subgroups and the national average for continuous variables (Step 1 and 2 scores, contiguous ranks, specialties ranked, research experiences, publications, and work and volunteer experiences). Chi-square tests were performed to test the significance of observed trends for qualitative variables (applicant type, AOA status, PhD, and other graduate degree) and to compare the difference among the categorical bins reported by the NRMP for quantitative continuous variables, as this allowed analysis of data in the most granular form possible given the manner in which the NRMP reports these outcomes. Statistical significance was set at p=0.05 for all analyses.

## Results

The average orthopaedic surgery match rate between 2016 and 2022 was 73.1%, though it has been declining since 2019 and fell to 62.3% in 2022 (Tables 1, 2). Data on applicant characteristics were available in the ICTO database for 57.9% of all applicants between 2018 and 2022, as reported by the Electronic Residency Application Service (ERAS) (Table 3) [19]. 

**Table 1:** Summary of match rate based on Interactive Charting Outcomes in the Match (ITCO) data for 2016-2022 as compared to the number of applicants reported by the Electronic Residency Application Service (ERAS) for 2018-2022.

Match Year	n Matched	n Unmatched	Total	% Matched	Number of Applicants (ERAS)	% Represented by ICTO Data
2016	531	203	734	72.3%	Not reported	Not reported
2017	553	174	727	76.1%	Not reported	Not reported
2018	612	171	783	78.2%	1242	63.0%
2019	611	160	771	79.2%	1321	58.4%
2020	663	233	896	74.0%	1401	64.0%
2021	598	245	843	70.9%	1502	56.1%
2022	538	325	863	62.3%	1714	50.4%
Total	4106	1511	5617	73.1%	7180	57.9%

**Table 2:** Summary of US senior applicant characteristics based on Charting Outcomes in the Match data for 2016, 2018, 2020, and 2022 with scores displayed as median (interquartile range).

Match Year	Total (n)	Step 1 Score	Step 2 Score	Mean Research Experiences	Mean Publications, Abstracts, Presentations	Mean Work Experiences	Mean Volunteer Experiences	Mean Contiguous Ranks
2016 Matched	622	248 (242-256)	254 (246-262)	4	8.2	3.2	6.7	12.1
2016 Unmatched	188	240 (228-248)	248 (236-254)	3.8	4.9	3.3	6.7	6.8
2018 Matched	678	248 (242-256)	256 (248-264)	4.9	11.5	3.2	7.3	12.5
2018 Unmatched	132	240 (232-252)	248 (238-256)	4.9	6.7	3.4	6.3	6.6
2020 Matched	645	248 (242-256)	252 (246-258)	5.4	14.3	3.6	8.0	12.3
2020 Unmatched	159	240 (230-250)	244 (232-252)	5.7	14.2	3.8	7.6	7.0
2022 Matched	574	248 (244-256)	256 (250-264)	6.6	16.5	4.0	8.9	12.2
2022 Unmatched	297	244 (234-252)	252 (242-258)	5.4	12.1	3.5	7.5	5.6

**Table 3:** Probability of matching with 95% confidence interval and risk difference compared to national average based on Step 1 score, Step 2 score, applicant type, number of research experiences, publications, work experiences, volunteer experiences, contiguous ranks, specialties ranked, PhD status, having obtained another graduate degree, and AOA membership status. AOA: Alpha Omega Alpha Honors Society; IMG: international medical graduate

	Category	n Matched	n Unmatched	% Match	95% CI	Risk Difference vs National Average	P value
Step 1 Score	No Score	2	1	66.7%	-	-6.4%	-
	<200	6	28	17.6%	12.8%	-55.5%	<0.001
	200-209	31	60	34.1%	9.7%	-39.0%	<0.001
	210-219	74	107	40.9%	7.2%	-32.2%	<0.001
	220-229	220	208	51.4%	4.7%	-21.7%	<0.001
	230-239	665	312	68.1%	2.9%	-5.0%	<0.001
	240-249	1242	432	74.2%	2.1%	1.1%	0.31
	250+	1866	363	83.7%	1.5%	10.6%	<0.001
Step 2 Score	<210	3	16	15.8%	16.4%	-57.3%	<0.001
	210-219	27	72	27.3%	8.8%	-45.8%	<0.001
	220-229	89	169	34.5%	5.8%	-38.6%	<0.001
	230-239	301	222	57.6%	4.2%	-15.5%	<0.001
	240-249	816	395	67.4%	2.6%	-5.7%	<0.001
	>250	2870	637	81.8%	1.3%	8.7%	<0.001
Applicant Type	US MD Senior	3634	965	79.0%	1.2%	5.9%	<0.001
	US DO Senior	225	118	65.6%	5.0%	-7.5%	0.003
	US IMG	37	100	27.0%	7.4%	-46.1%	<0.001
	Non-US IMG	25	94	21.0%	7.3%	-52.1%	<0.001
	Prior US MD Grad	177	214	45.3%	4.9%	-27.8%	<0.001
	Prior US DO Grad	8	20	28.6%	16.7%	-44.5%	<0.001
Research Experiences	None	27	28	49.1%	13.2%	-24.0%	<0.001
	<3	713	369	65.9%	2.8%	-7.2%	<0.001
	3-5	2136	734	74.4%	1.6%	1.3%	0.10
	5-10	969	284	77.3%	2.3%	4.2%	<0.001
	11-15	152	50	75.2%	6.0%	2.1%	0.48
	16-20	60	20	75.0%	9.5%	1.9%	0.69
	21-25	17	5	77.3%	17.5%	4.2%	0.64
	≥25	32	12	72.7%	13.2%	-0.4%	0.96
Publications	None	148	104	58.7%	6.1%	-14.4%	<0.001
	< 3	464	249	65.1%	3.5%	-8.0%	<0.001
	3-5	889	369	70.7%	2.5%	-2.4%	0.06
	5-10	1091	335	76.5%	2.2%	3.4%	0.002
	11-15	562	154	78.5%	3.0%	5.4%	<0.001
	16-20	302	95	76.1%	4.2%	3.0%	0.17
	21-25	167	57	74.6%	5.7%	1.5%	0.62
	≥25	483	148	76.5%	3.3%	3.4%	0.04
Work Experiences	None	168	78	68.3%	5.8%	-4.8%	0.11
	< 3	1289	456	73.9%	2.1%	0.8%	0.46
	3-5	1987	682	74.4%	1.7%	1.3%	0.11
	5-10	606	261	69.9%	3.1%	-3.2%	0.04
	11-15	48	22	68.6%	10.9%	-4.5%	0.41
	16-20	6	8	42.9%	25.9%	-30.2%	0.02
	21-25	2	1	66.7%	-	-6.4%	-
	≥25	0	3	0.0%	-	-73.1%	-
Volunteer Experiences	None	22	19	53.7%	15.3%	-19.4%	0.013
	< 3	234	160	59.4%	4.8%	-13.7%	<0.001
	3-5	1186	512	69.8%	2.2%	-3.3%	0.003
	5-10	1859	612	75.2%	1.7%	2.1%	0.01
	11-15	617	161	79.3%	2.8%	6.2%	<0.001
	16-20	135	34	79.9%	6.0%	6.8%	0.03
	21-25	40	6	87.0%	9.7%	13.9%	0.01
	≥25	13	7	65.0%	20.9%	-8.1%	0.45
PhD Status	PhD degree	49	27	64.5%	10.8%	-8.6%	0.12
Other Graduate Degree	Non-PhD graduate degree	669	363	64.8%	2.9%	-8.3%	<0.001
AOA Status	AOA member	1480	152	90.7%	1.4%	17.6%	<0.001
Contiguous Ranks	<3	143	429	25.0%	3.5%	-48.1%	<0.001
	3-5	404	512	44.1%	3.2%	-29.0%	<0.001
	5-10	1222	407	75.0%	2.1%	1.9%	0.07
	11-15	1339	120	91.8%	1.4%	18.7%	<0.001
	16-20	786	40	95.2%	1.5%	22.1%	<0.001
	21-25	172	2	98.9%	1.6%	25.8%	<0.001
	>25	40	0	100.0%	-	26.9%	-
Specialties Ranked	1	3791	1054	78.2%	1.2%	5.1%	<0.001
	2	285	371	43.4%	3.8%	-29.7%	<0.001
	3	23	62	27.1%	9.4%	-46.0%	<0.001
	4	7	20	25.9%	16.5%	-47.2%	<0.001
	5+	0	4	0.0%	-	-73.1%	-

USMLE scores

Individuals with Step 1 score less than 240 had a decreased probability of matching (p<0.01), those with Step 1 score 240-249 did not have a significantly different probability of matching (p=0.31), and applicants with a Step 1 score of ≥250 had a 10.6% increased probability of matching into orthopaedic surgery compared to national average (p<0.01). A Step 2 score of 249 or below was associated with a decreased match rate compared to the national average (p<0.01), while a Step 2 score ≥250 was associated with an 8.7% increased probability of matching (p<0.01).

Applicant type

The US MD applicants had a 5.9% increased probability of matching compared to the national average for all applicants (p<0.01), while US DO seniors, prior US MD graduates, prior US DO graduates, US International medical graduates, and non-US medical graduates had a decreased probability of matching compared to the national average. The NRMP did not provide any ICTO data regarding possible applicants through the Canadian or US fifth pathway applicants during this time frame.

Graduate degree and AOA status

AOA Honors Society members had a 17.6% increased probability of matching compared to the national average for all applicants (p<0.01). Applicants with non-PhD graduate degrees had decreased probabilities of matching (p<0.01), and applicants with PhD degrees had no statistically significant change in match probability from the average applicant though a decreasing trend was noted (p=0.12).

Research experiences and publications

**Figure 1:**
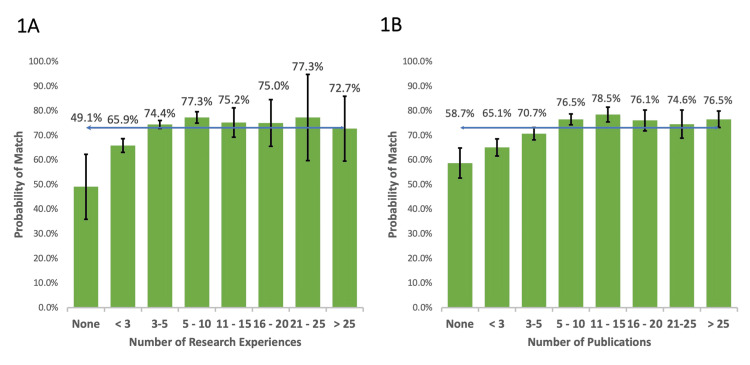
Probability of matching based on number of research experiences (a) and publications (b) compared to average match rate for all applicants of 73.1% (blue line).

Applicants with fewer than three research experiences had a decreased probability of matching compared to the national average. Applicants with five to 10 research experiences had a 4.2% increased probability of matching (p<0.01) while those with ≥11 research experiences did not have a statistically significant increased probability of matching compared to the national average (Figure 1A). Subsequent comparative analysis confirmed that individuals with five to 10 research experiences had a higher probability of match compared to those with three to five research experiences (p=.046), but any number of experiences beyond five did not increase the probability of matching (p=.361). 

Applicants with less than three publications had a decreased probability of matching while those with three to five or 15-20 publications did not have a significantly different probability of matching compared to the national average (Figure 1B). Applicants with 5-10, 11-15, or ≥25 publications had an increased probability of matching as compared to the national average with comparative analysis subsequently showing no significant change in match probability once five or more publications were obtained (p=0.773).

Work and volunteer experiences

**Figure 2:**
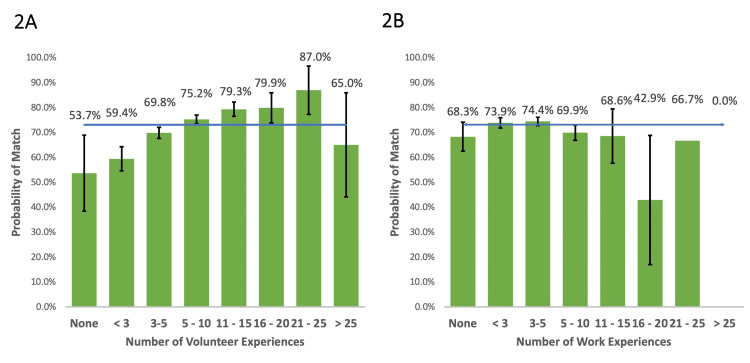
Probability of matching based on number of volunteer experiences (a) and work experiences (b) compared to average match rate for all applicants of 73.1% (blue line).

Applicants with less than five or ≥25 volunteer experiences had a decreased probability of matching while applicants with five to 25 experiences had an increased probability of matching compared to the national average. The probability of matching increased with additional volunteer experiences through 21-25 volunteer experiences (Figure 2A). Applicants with five to 10 or 16-20 work experiences had a decreased probability of matching compared to the national average, while applicants with less than five or 11-15 experiences did not have a statistically significant different probability of matching (Figure 2B). The sample size for 21-25 and ≥26 work experiences was too small (n<5) to compare to the national average. Subsequent comparative analysis showed that applicants with more than five work experiences had a decreased probability of matching compared to applicants with three to five work experiences (p=0.002), though applicants with five to 10 experiences did not have a significantly lower probability of matching than those with ≥11 experiences (p=0.133).

Contiguous ranks and distinct specialties

**Figure 3:**
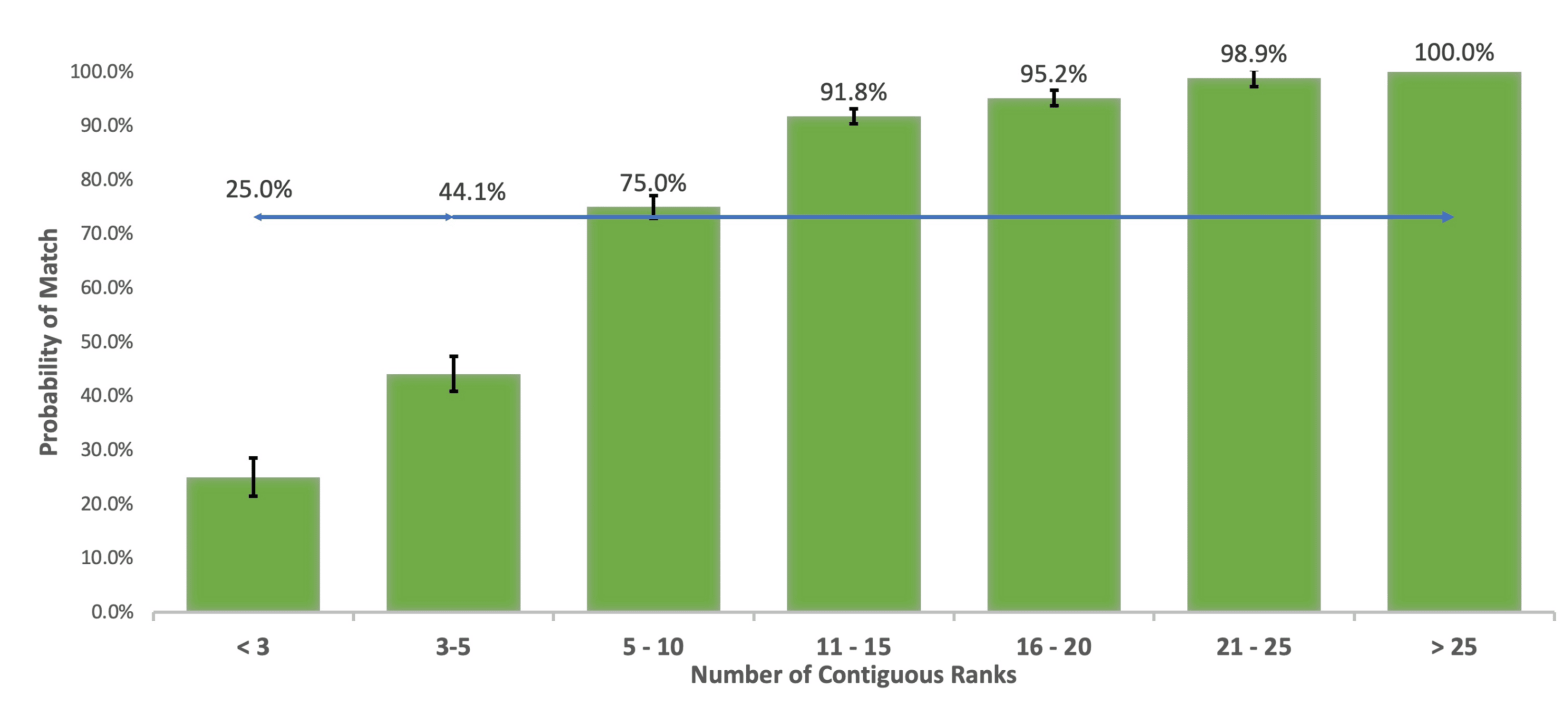
Probability of matching based on number of continuous ranks compared to average match rate for all applicants of 73.1% (blue line).

Applicants with fewer than five contiguous ranks had a decreased probability (p<0.01), those with five to 10 contiguous ranks did not have a probability significantly different from the national average (p =0.07), and applicants with ≥11 contiguous ranks had a higher probability of matching compared to the national average (p<0.01). Match probability continued to increase as the number of contiguous ranks increased (Figure 3). Subsequent comparative analysis demonstrated applicants with >20 contiguous ranks had a higher probability of matching compared to applicants with 15-20 contiguous ranks (p<0.01), though there was insufficient data to compare applicants with ≥26 contiguous ranks to applicants with 20-25 contiguous ranks. Of note, data on the number of contiguous ranks is based on the number of ranks in that specialty. Ranking residency programs in more than one specialty was associated with a decreased probability of matching (Table 3: one specialty=78.2%, two specialties=43.4%, three specialties=27.1%, four specialties=25.9%, five or more specialties=0.0%). 

Match rate was compared based on the number of contiguous ranks for the applicants between 2016 and 2020 with traditional interviews versus applications between 2021 and 2022 where interviews were almost exclusively virtual (Table 4). The match rate was overall similar for applicants between the two cohorts based on the number of ranks; however, all 82 applicants (100%) in 2021-2022 who ranked 21 or more programs matched successfully.

**Table 4:** Comparison of match rate based on number of contiguous ranks for 2016-2020 versus 2021-2022 applicants.

		2016-2020			2021-2022		
		n Matched	n Unmatched	% Matched	n Matched	n Unmatched	% Matched
Contiguous Ranks	<3	97	266	26.7	46	163	22.0
	3-5	277	304	47.7	127	208	37.9
	5-10	843	257	76.6	379	150	71.6
	11-15	1028	80	92.8	311	40	88.6
	16-20	595	31	95.0	191	9	95.5
	21-25	111	2	98.2	61	0	100.0
	>25	19	0	95.0	21	0	100.0
	Total	2970	940	76.0	1136	570	66.6

## Discussion

Matching into an orthopaedic surgery residency is increasingly difficult, with an average match rate of 66.6% in 2021-2022 compared to 76.0% over 2016-2020 despite recent efforts to make the NRMP more applicant-centric and improve the equitability of the match process [20,21]. The results of this study show that applicants who possess higher USMLE scores, have obtained AOA membership, are US MD applicants, and rank ≥11 programs within orthopaedics alone are more likely to successfully match. Paradoxically, having a non-PhD graduate degree was associated with a decreased probability of matching, and having a PhD resulted in a down-trending, though not statistically significant, change in match rate. 

We found a continuing significant increase in the probability of matching until over 20 programs are ranked (Figure 3). This finding is of particular interest as it contradicts the common discouragement of “interview hoarding” since interviews were made virtual. Manjunath and Morrill explained in their 2021 analysis that virtual interviews remove the two greatest constraints for accepting as many interviews as possible: time and money [22]. As a result, top-tier applicants interview at many programs - even those in which they are unlikely to rank highly - while excellent but slightly lower-tier applicants are prevented from interviewing. While some authors have suggested programs will increase their number of interviews to avoid a decreasing trend in the percentage of filled positions, others suggest the NRMP will be forced to either limit the number of interviews that can be accepted or increase transparency regarding interviews accepted so programs can select applicants with a reasonable chance of ranking their program [22,23]. However, in the current environment, excluding interview limitation or increased transparency, it appears accepting as many interviews as possible is of individual benefit. Further study is necessary to better understand this trend and regulate the potentially conflicting interests of individual applicants versus applicants as a group. 

Our study found a continued correlation between successful match and high USMLE performance, consistent with past studies demonstrating that Step 1 score was among the most important residency selection criteria along with completing an away rotation, class rank, personability, and letters of recommendation [6,24]. However, several studies have found USMLE Step 1 scores to be weakly predictive of the ability to succeed as an orthopaedic resident or performance on the Orthopaedic In-Training Examination (OITE) [5,25]. Similarly, Dirschl et al. [26] and Calhoun et al. [27] found medical school third-year clerkship grades to be the most valuable predictors while Step 1 scores had no correlation to resident performance. Though the Step 1 score will be less important among selection criteria since the exam became pass/fail in 2022, our results show that the Step 2 score serves as another significant criterion, and its importance will likely increase in the absence of a numerical Step 1 score. Additionally, Camp et al. found AOA status, research productivity, and standardized test scores lack correlation with skills such as self-awareness, moral integrity, dexterity and work ethic [28], which arguably are more valuable qualities of an excellent orthopaedic resident [5]. Ultimately, though imperfect predictors of resident success, board scores, and academic performance during medical school correlate to successfully matching, likely representing the challenging and subjective task before faculty of surgical subspecialty residency programs in the absence of a predetermined formula [5,29,30].

Diversity, equity and inclusion (DEI) within medicine is critical to patient care outcomes [31] and specifically orthopaedic surgery outcomes [32]. Though orthopaedic surgery continues to fall behind other medical specialties with respect to diversity [33], DEI efforts were recently named a top priority for orthopaedics [34]. The results of this study pose several interesting questions with regards to resident selection and DEI efforts. We found AOA status, traditionally offered to students with demonstrated leadership, teaching ability, academic prowess, and service to one’s medical school, increased the chances of a successful match by approximately 17% compared to the national average. However, the value of this metric may be compromised by the number of schools without AOA chapters [5] and recently found inequitable demographic distribution of AOA status even after controlling for many of the aforementioned characteristics [35]. This subsequently called many institutions to reform or suspend AOA elections until national changes are put into effect [36]. Additionally, we found an increased number of work experiences (more than five) correlated with a decreased chance of matching (Figure 2A), which may negatively impact applicants from under-privileged backgrounds whose financial circumstances required working rather than volunteering, as well as non-traditional medical students who held previous careers, among other potential impacts. Furthermore, we found that additional PhD or non-PhD graduate degrees either decreased or trended towards decreasing one’s chance of matching. While on the one hand, this may negatively impact academically prodigious students who sought to attain additional post-graduate degrees, it may also disadvantage under-privileged applicants who may have obtained an additional degree to bolster their undergraduate GPA when applying to medical school, given that 43% of the AAMC’s collated post-baccalaureate programs report focus on under-represented minority students [37]. Ultimately, few conclusions can be drawn given the limitations of the current publicly available data, but these findings may prompt institutions to ensure their evaluation process is not at odds with their DEI efforts in the future. A follow-up study involving interviews with program directors may help to better understand these trends.

Our findings provide students insight into maximizing their probability of matching. Both research experiences and publications positively influence the probability of a successful match (Figure 1). However, our chi-square analysis suggests no added benefit beyond five research experiences and 10 publications. This may represent program directors evaluating research experience quality over quantity or simply the inability of a student to obtain, for example, >15 publications and remain well-rounded in other valuable aspects of their application. Therefore, our interpretation of this data is that students may consider taking on a manageable number of research projects they could passionately discuss in a residency interview as opposed to maximizing the number of projects at the expense of quality. However, an assumption in this recommendation is that applicants would not be able to achieve a high volume of high-quality research within the confines of a standard four-year medical school curriculum. Applicants with extensive research backgrounds, either via additional graduate studies or dedicated research time, may have unique circumstances where these generally observed trends do not apply. 

Applicants may additionally consider focusing their time on volunteer efforts, as demonstrated by the positive relationship between volunteer experiences and match rate (Figure 2). Our research also supports students attempting to obtain AOA status if they are enrolled in a school with an active chapter. Graduate degrees (both PhDs and other non-PhD graduate degrees) do not appear to increase the probability of matching; however, a decision to pursue graduate degrees is multifaceted.

Our study has several limitations. First, we reviewed pooled data representing macro trends. Though primarily a limitation of the NRMP ICTO data platform, we could not complete a comprehensive, multivariate analysis based on free, publicly accessible data. Second, we were unable to analyse differences between gender or ethnicity as the NRMP tool did not report this data. Third, our analysis fails to include an unknown number of applicants who opted out of being included in the NRMP ICTO database, which may be as high as 42.1% of applicants based on a comparison of NRMP tool data to all orthopaedic residency positions [8]. However, a similar limitation in the percentage of students reporting results is present in the widely cited and biannually published Charting the Outcomes reports [38-45], and NRMP data remain the gold standard for applicants to review and, by extension, make decisions regarding their experiences and, chances prior to applying for residency. Fourth, though our findings indicated an increasing likelihood of successful match with a number of contiguous ranks, it is possible for applicants to interview and not rank a program (though this essentially conveys to the program that this applicant would prefer not to match), and there is an additional $30 fee incurred for each program ranked after 20 programs [46]. Overall, we feel that the number of contiguous ranks is an appropriate proxy for the number of interviews but may be less accurate for evaluating >20 programs ranked. Fifth, though our results found an increasing match rate with Step 2 score ≥250, we were unable to further stratify scores between 250 and 265 and beyond 265 due to NRMP data presentation. Similarly, due to data presentation, the groups of contiguous variables analyzed, such as a Step 2 score of 235-250, prevent recognition of small differences in match rate, such as a Step 2 score of 235 versus 240. Finally, the NRMP ICTO tool cannot consider letters of recommendation, interview success, interpersonal connections, and other qualitative data that are highly regarded in applicant selection [5].

## Conclusions

The orthopaedic surgery residency match remains increasingly competitive, and programs are challenged now more than ever to filter through highly qualified applicants. By highlighting applicant-level trends through aggregate analysis of NRMP data, this study clarifies what factors are most beneficial to a successful match so students can best prioritize their efforts before applying to residency. Most notably, our results showed a small but significant increase in the chance of matching with a number of contiguous ranks ≥11, which poses a challenge between individual benefit and the national match as a whole. Our study findings would support an individual applicant accepting interviews to and ranking as many programs as possible to increase the probability of matching. In addition, despite previous studies suggesting a poor correlation with residency performance, obtaining five to 10 publications, seeking AOA status, gaining volunteer experiences, and performing well on USMLE exams may benefit students as they apply for orthopaedic surgery residency based on our findings. Additional graduate degrees did not improve the probability of matching in our analysis. Ultimately, this study provides a guide as students seek to craft a residency application that will have the greatest chance of a successful match into orthopaedic surgery.
